# Indirect Transmission of Influenza A Virus between Pig Populations under Two Different Biosecurity Settings

**DOI:** 10.1371/journal.pone.0067293

**Published:** 2013-06-21

**Authors:** Matt W. Allerson, Carol J. Cardona, Montserrat Torremorell

**Affiliations:** 1 Department of Veterinary Population Medicine, College of Veterinary Medicine, University of Minnesota, St. Paul, Minnesota, United States of America; 2 Department of Veterinary and Biomedical Sciences, College of Veterinary Medicine, University of Minnesota, St. Paul, Minnesota, United States of America; University of Georgia, United States of America

## Abstract

Respiratory disease due to influenza virus is common in both human and swine populations around the world with multiple transmission routes capable of transmitting influenza virus, including indirect routes. The objective of this study was to evaluate the role of fomites in influenza A virus (IAV) transmission between pig populations separated by two different biosecurity settings. Thirty-five pigs were divided into four experimental groups: 10 pigs (1 replicate) were assigned to the infected group (I), 10 pigs (2 replicates of 5 pigs) were assigned to the low biosecurity sentinel group (LB), 10 pigs (2 replicates of 5 pigs) were assigned to the medium biosecurity sentinel group (MB), and 5 pigs (1 replicate) were assigned to the negative control group (NC). Eight of 10 pigs in the infected group were inoculated with IAV and 36 hours following inoculation, personnel movement events took place in order to move potentially infectious clothing and personal protective equipment (PPE) to sentinel pig rooms. Following contact with the infected group, personnel moved to the MB group after designated hygiene measures while personnel moved directly to the LB group. Nasal swabs and blood samples were collected from pigs to assess IAV infection status and fomites were sampled and tested via RRT-PCR. All experimentally inoculated pigs were infected with IAV and 11 of the 144 fomite samples collected following contact with infected pigs were low level positive for IAV genome. One replicate of each sentinel groups LB and MB became infected with IAV and all five pigs were infected over time. This study provides evidence that fomites can serve as an IAV transmission route from infected to sentinel pigs and highlights the need to focus on indirect routes as well as direct routes of transmission for IAV.

## Introduction

During the period of 1976-2007, 1.4 to 16.7 deaths per 100,000 persons were influenza-associated in the United States each year [1]. In addition to the significant mortality and morbidity associated with influenza virus in the human population, influenza A virus (IAV) is a common pathogen in many animal species, including pigs. Influenza virus has been considered widespread in the United States pig population since first described clinically in 1918 [2]. Classical H1N1 viruses were the dominant circulating influenza viruses in pigs in the United States until the appearance and subsequent circulation of triple reassortant H3N2 viruses in 1998, leading to a more complex epidemiologic picture [3].

While many different IAV subtypes and genotypes have been described in pigs and circulate today, common transmission routes exist and transmission of IAV can occur via several different routes. In addition to direct contact with infected hosts, aerosols and fomites may serve as transmission routes for IAV [4]. These transmission routes are not only applicable for within species transmission, but they are also important for interspecies transmission. In 2009, an H1N1 influenza virus with gene segments of swine lineage and a gene combination not previously reported was detected in humans in North America [5]. This virus ultimately infected humans across the globe and became widespread in animal populations, including pigs. The 2009 pandemic H1N1 and H3N2 variant (H3N2v) viruses [6], [7] have recently highlighted the role of interspecies transmission in IAV epidemiology.

While it is known that direct pig to pig transmission of IAV occurs, other transmission routes have not been fully elucidated. Indirect transmission routes, such as contaminated personnel or fomites, have been shown to transmit other pathogens from infected to susceptible pigs [8], [9]. Influenza viruses have been recovered from fomites and the hands of people [10]-[12]; however, limited information exists regarding the subsequent infection of susceptible hosts from contaminated fomites. Furthermore, biosecurity measures (e.g. hand washing and wearing clean outerwear and boots) are commonly utilized by animal and public health personnel between different populations or visits to prevent IAV transmission, but there is limited information on the effectiveness of these measures. The objective of this study was to evaluate the role of fomites in IAV transmission between pig populations separated by two different biosecurity settings and this was accomplished using a pig challenge and exposure model.

## Materials and Methods

### Ethics statement

All pigs were monitored daily and cared for according to the University of Minnesota Animal Care and Use Protocol number 1109A05201. This study and the Animal Care and Use Protocol were specifically approved by the University of Minnesota Institutional Animal Care and Use Committee (IACUC). Due to the use of an infectious agent (influenza A virus), this study was also approved by the University of Minnesota Institutional Biosafety Committee (IBC), protocol number 1109H04982. Personnel wore the following clothing and personal protective equipment (PPE) when in contact with pigs: coveralls, boots, bouffant cap, protective eyewear, N-95 respirator, and gloves. Fomite sample collection was from clothing and PPE, not from study personnel directly. In addition, fomite samples were collected to assess whether IAV could be detected from the specific fomites and results were not linked to a specifically named person. The interaction with pigs simulated contact normally encountered in pig facilities. Human subject approval was therefore not applicable and was not obtained for this study; however, personnel provided verbal consent to participate in the study with the process documented as part of regular planning meetings prior to the start of the study. Personnel were also approved to participate as part of the aforementioned approved IACUC and IBC protocols.

### Animals and animal housing

Thirty-five pigs (average age of 6 weeks) were assigned to one of four experimental groups. Ten pigs (1 replicate) were assigned to the infected group (I), 10 pigs (2 replicates of 5 pigs) were assigned to the low biosecurity sentinel group (LB), 10 pigs (2 replicates of 5 pigs) were assigned to the medium biosecurity sentinel group (MB), and 5 pigs (1 replicate) were assigned to the negative control (NC) group (Table 1). All pigs used in this study tested negative for IAV antibodies at the source herd and 5 days prior to the start of the study after movement to the University of Minnesota animal isolation facility. Serum samples were tested via enzyme linked immunosorbent assay (ELISA) (FlockChek® Avian Influenza MultiS-Screen Antibody Test Kit, IDEXX Laboratories Inc., Westbrook, ME, USA) as described previously [13]. Nasal swabs collected from all pigs 5 days prior to the start of the study also tested negative for IAV RNA via a matrix gene based real-time reverse transcription PCR (RRT-PCR) [14]. A convenience selection of 9 serum samples from pigs used in this study were determined to be negative for porcine reproductive and respiratory syndrome virus (PRRSv) (HerdChek® PRRS Antibody X3 Test Kit, IDEXX Laboratories Inc., Westbrook, ME, USA) and *Mycoplasma hyopneumoniae* (HerdChek® *M. hyopneumoniae* Antibody Test Kit, IDEXX Laboratories Inc., Westbrook, ME, USA*)* antibodies. In addition, the source herd was determined to be free of PRRSv and *Mycoplasma hyopneumoniae* by historical antibody testing.

**Table 1:** Experimental groups.

Group	Group code	N	Replicates	Isolation room	Person in contact
Infected	I	10	1	1	A, B, C, D
Low biosecurity sentinel	LB	10	2 (n = 5)	2 and 4	A, B
Medium biosecurity sentinel	MB	10	2 (n = 5)	3 and 5	C, D
Negative control	NC	5	1	6	Other personnel

**Figure 1:**
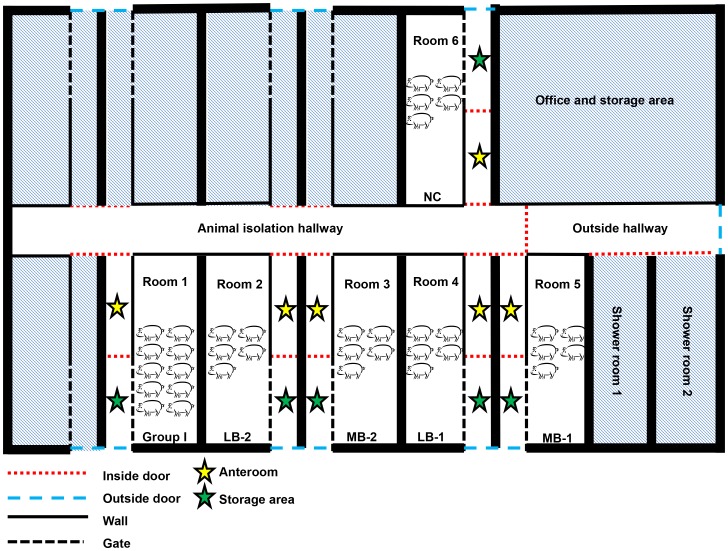
Isolation facility layout.

All pigs were housed at the University of Minnesota animal isolation facility (St. Paul, MN, USA) with each replicate placed in a separate isolation room (Figure 1). The isolation barn had 11 individual animal isolation rooms (6 used for this study) with a shared hallway. Each animal isolation room had an anteroom with footbaths, a sink for hand washing, and a storage area for equipment. In addition, each animal isolation room had a storage area of 2.08 m^2^ and one animal housing area of 7.28 m^2^ (1.5 m^2^/pig in sentinel pig rooms). The floor of the animal housing area was solid concrete and the housing area had one water line with two water nipples. All rooms had negative pressure ventilation systems with one air inlet and one air outlet and the total airspace volume of each room was 35.1 m^3^. The incoming air to each room was filtered with a 3 ply panel filter (TRI-DEK® 15/40, TRI-DIM Filter Corp., Louisa, VA, USA) and exhaust air was filtered with a HEPA filter (XH Absolute HEPA filter, Camfil Farr, Inc., Stockholm, Sweden).

### Experimental groups

#### Infected group (I)

Eight pigs were challenged intra-tracheally and intra-nasally with 1 mL of viral inoculum in each location, containing 4.6×10^6^ tissue culture infective dose (TCID_50_ */*mL) of a delta cluster H1N1 influenza A virus (A/Sw/MN/07002083/07). The virus was originally isolated at the University of Minnesota Veterinary Diagnostic Laboratory from an outbreak of respiratory disease in pigs and has been used previously [15]. The viral isolate was grown in bulk quantities using Madin-Darby canine kidney (MDCK) cells [16]. Before the challenge inoculation, all pigs were sedated by an intramuscular injection of Telazol® (6 mg/kg, Telazol®, Fort Dodge Animal Health, Fort Dodge, IA, USA). Two pigs in this group (direct contact sentinels) were not inoculated and were moved to a separate isolation room prior to the challenge virus inoculation. Twenty-four hours later, the direct contact sentinel pigs were moved back with the 8 experimentally inoculated pigs.

#### Low biosecurity sentinel group (LB)

Ten IAV negative pigs were placed in two separate isolation rooms (5 pigs/room, 2 replicates). Two study personnel (personnel A and B) each moved directly from group I to one of the LB replicates. The same person moved to the same replicate for all nine movement events with the exception of a replacement for person A at movement event 1.

#### Medium biosecurity sentinel group (MB)

Ten IAV negative pigs were placed in two separate isolation rooms (5 pigs/room, 2 replicates). Two study personnel (personnel C and D) each performed a series of biosecurity measures before moving to one of the MB replicates. The same person moved to the same replicate for all nine movement events.

#### Negative control group (NC)

Five IAV negative pigs were placed in an isolation room. All personnel caring for this group did not have contact with the I, LB, or MB groups during the entire movement period.

### Study personnel

Study personnel had no other direct pig contact for the duration of this study. Personnel A and B had direct contact with pigs in groups I and LB and personnel C and D had direct contact with pigs in groups I and MB during the course of the movement period. Isolation facility personnel and other personnel that did not have contact with pigs in groups I, LB, or MB cared for pigs in group NC. Personnel A, B, C, and D performed all necessary procedures (e.g. pig nasal swab collection, feeding of pigs, cleaning rooms) in their respective sentinel rooms in order to prevent entry of other personnel into rooms during the movement period and the sampling dates thereafter.

### Clothing and personal protective equipment (PPE)

All personnel wore the same clothing and PPE, except person A wore cloth coveralls at all times in contrast to Tyvek® coveralls worn by personnel B, C, and D (Table 2). Person A wore cloth coveralls so that both types of coveralls could be assessed in the LB setting where coveralls were not changed prior to movement to sentinel rooms. Due to the potential for interspecies transmission and to comply with approved University of Minnesota Institutional Biosafety Committee protocol 1109H04982, personnel were required to wear PPE in addition to boots, coveralls, and gloves commonly worn by study and farm personnel. The PPE included a bouffant cap, protective eyewear, and N-95 respirator.

**Table 2:** Clothing and personal protective equipment (PPE).

Clothing or PPE	Manufacturer
Undershirt and pants	Various manufacturers (55% cotton, 45% polyester)
Tyvek® coverall	DuPont™ Tyvek®, Wilmington, DE, USA
Cloth coverall	Various manufacturers (65% polyester, 35% cotton)
Rubber boots	Tingley Rubber Corp., South Plainfield, NJ, USA
Disposable plastic boots	KNOT-a-BOOT™, Continental Plastic Corp., Delavan, WI, USA
Polypropylene bouffant cap	Medline Industries Inc., Mundelein, IL, USA
Protective eyewear	MSA, Safety Works®, Cranberry Township, PA, USA
N-95 respirator	3M™ (9210/37021), St. Paul, MN, USA
Powder-free latex gloves	Microflex® Evolution One®, Reno, NV, USA

Before entry into the group I room, personnel showered in the isolation facility. Following the shower procedure, personnel placed on an undershirt and pants and a pair of disposable plastic boots and entered the animal isolation hallway after stepping in an iodine footbath. In the animal isolation hallway, personnel placed on coveralls, latex gloves, a bouffant cap, and transported a pair of disposable plastic boots to the group I room. Upon entry to the group I anteroom, personnel stepped in an iodine footbath and placed on a N-95 respirator, room specific rubber boots, and room specific protective eyewear. Before entering the animal housing area, personnel placed on a pair of disposable plastic boots over their rubber boots.

### Movement between experimental groups

**Figure 2:**
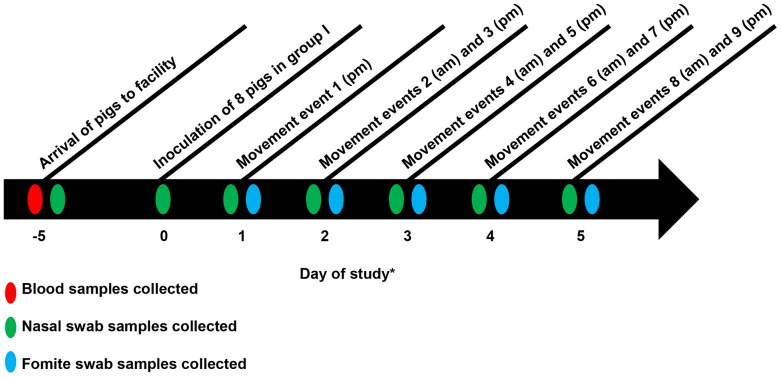
Timeline following arrival and during movement events. *In relation to day of inoculation.

A movement event was defined as the movement of personnel from group I to groups LB and MB. There were a total of 9 movement events over a 5 day period for each LB and MB replicate. The first movement event took place in the afternoon, approximately 36 hours following the experimental inoculation of pigs in group I (Figure 2). Movement events then took place during the morning (am) and afternoon (pm) of 4 consecutive days. Therefore, the second movement event (am) followed the first movement event (pm) by approximately 16 hours, the third movement event (pm) followed the second movement event by approximately 8 hours. Movement event duration was timed according to the estimated infectious period (1 to 5 days post-inoculation) of pigs in group I, and confirmed by sampling pigs in group I daily.

#### Exposure of personnel to infected and sentinel pigs

For each of the 9 movement events, 4 study personnel (A, B, C, and D) were exposed to pigs in group I at the same time for a period of 45 minutes after following procedures outlined above in the clothing and PPE section. Pigs were allowed to have direct contact with clothing and PPE for the 45 minute period. Personnel spent the 45 minute period in the infected pig room standing and sitting on the floor while handling pigs as they moved throughout the room. Therefore, pigs had access to all clothing and PPE worn by personnel. This period allowed for thorough exposure to potentially infectious secretions. All pigs in group I interacted with all study personnel during the exposure period and all personnel interacted in a similar manner with the infected pigs. Following movement of personnel to sentinel groups LB and MB, the same exposure period of 45 minutes was repeated to allow for sentinel pigs in groups LB and MB to contact potentially infectious clothing and PPE.

#### Movement of personnel A and B to LB rooms

Following the 45 minute interaction period of personnel A and B with group I pigs, personnel A and B placed their used coveralls, disposable plastic boots, latex gloves, and bouffant cap in a clean plastic bag in the group I storage area. Personnel A and B exited the group I room via the outside door and entered their respective LB sentinel group storage area through the outside door and placed on the used coveralls, disposable plastic boots, gloves, and bouffant cap. Each person collected four separate fomite swab samples from (1) coveralls, (2) disposable plastic boots, (3) latex gloves, and (4) the bouffant cap and outer surface of the N-95 respirator in the group LB storage area. Following the fomite swab sample collection, personnel interacted with sentinel group LB pigs (Table 3). Personnel A and B did not wash their hands or face during this process.

**Table 3:** Clothing and PPE changed after contact with group I.

Clothing or PPE	Movement to LB group	Movement to MB group
Undershirt and pants	No	No
Coverall	No	Yes
Rubber boots	Yes (room specific)	Yes (room specific)
Disposable plastic boots	No	Yes
Polypropylene bouffant cap	No	Yes
Protective eyewear	Yes (room specific)	Yes (room specific)
N-95 respirator	No	Yes
Powder-free latex gloves	No	Yes

#### Movement of personnel C and D to MB rooms

Following the 45 minute interaction period of personnel C and D with group I pigs, each person collected four separate swab samples from (1) coveralls, (2) disposable plastic boots, (3) latex gloves, and (4) the bouffant cap and outer surface of the N-95 respirator in the group I storage area. Personnel C and D entered the group I anteroom and disposed of their used coveralls, disposable plastic boots, latex gloves, bouffant cap, and N-95 respirator. Personnel washed their hands and face with soap and water for approximately 40-60 seconds according to World Health Organization (WHO) guidelines and moved towards the MB sentinel rooms through the animal isolation hallway. In the anteroom of the MB sentinel rooms, personnel C and D washed their hands and face again and placed on new coveralls, a new pair of latex gloves, a new bouffant cap, room specific rubber boots, a new pair of disposable plastic boots, a new N-95 respirator, and room specific protective eyewear (Table 3). Each person collected four separate swab samples from (1) coveralls, (2) disposable plastic boots, (3) latex gloves, and (4) the bouffant cap and outer surface of the N-95 respirator in order to ensure that the new materials were negative for IAV following the biosecurity procedures. Following the fomite swab sample collection, personnel C and D interacted with sentinel group MB pigs.

### Sample collection, processing, and testing

#### Fomite swabs

For all fomite and nasal swab samples, a sterile rayon-tipped swab was used (BD BBL™ CultureSwab™, liquid Stuart medium, single plastic applicator, Becton, Dickinson and Co., Sparks, MD, USA). The chest area, front and back of each leg, and front and back of each arm were swabbed in a zigzag pattern for each coverall sample. For the boot sample, the entire surface of each disposable plastic boot was swabbed via a zigzag pattern in addition to visibly contaminated areas of the boot surface. The entire surface of each latex glove was swabbed and the outer surfaces of the bouffant cap and N-95 respirator were swabbed. Following collection and transport, each swab was suspended in 1.8 mL of brain–heart infusion (BHI) medium. Samples were tested for IAV via a matrix gene based real-time reverse transcription PCR (RRT-PCR) [14]. Cutoff Ct values for the RRT-PCR assay used in this study were: ≤35 positive, >35 and ≤40 low level positive or suspect, and >40 negative. In addition, all fomite samples in which IAV RNA was detected were tested by virus isolation on MDCK cell monolayers.

#### Nasal swabs

Nasal swabs were collected from all pigs prior to the start of the study and following inoculation of pigs in group I (Figure 2). Bilateral nasal swabs were collected using sterile rayon-tipped swabs and placed in liquid Stuart medium. Following collection and transport, each nasal swab was suspended in 1.8 mL of brain–heart infusion (BHI) medium. Samples were tested for IAV via matrix gene RRT-PCR. In addition, one nasal swab sample from each pig in the infected group (I) was tested by virus isolation and virus titration on MDCK cell monolayers with TCID_50_/mL calculated by the method of Spearman–Karber. The nasal swab samples tested by virus isolation and virus titration were from 2 days post inoculation (DPI) from the inoculated pigs (n = 8) and 5 DPI from the direct contact sentinels (n = 2). Hemagglutinin (HA) gene sequences were obtained from positive nasal swab samples from each infected pig in the LB and MB groups (n = 10), the inoculum (n = 1), and two positive samples from two different pigs in group I using previously described specific primers for HA [17] at the University of Minnesota Veterinary Diagnostic Laboratory. HA1 gene sequences obtained from all groups were compared to ensure that there were no new virus introductions.

#### Blood samples

Blood samples were collected via jugular venipuncture and serum was separated and stored at −20°C until testing. Samples were collected from all pigs prior to the start of the study at -5 DPI. Samples were also collected from pigs prior to euthanasia (15 DPI for group I and 21–28 DPI for the remainder of the pigs). Samples were tested for IAV antibodies via enzyme linked immunosorbent assay (ELISA) (FlockChek® Avian Influenza MultiS-Screen Antibody Test Kit, IDEXX Laboratories Inc., Westbrook, ME, USA) as described previously with an S/N ratio ≤ 0.673 considered positive and an S/N ratio > 0.673 considered negative [13]. The Influenza A Multiscreen ELISA measures antibodies directed against the nucleoprotein (NP) of influenza A viruses.

### Statistical Analyses

Statistical analyses were performed using SAS (SAS System, SAS Inst., Cary, North Carolina, v 9.2) and R (R Foundation for Statistical Computing, Vienna, Austria). ELISA antibody titers at -5 DPI and prior to euthanasia were compared via Student’s paired t-test. Hemagglutinin gene sequences were aligned and compared using the ClustalW algorithm using MegAlign™ software (DNASTAR, Inc., Madison, WI, USA).

## Results

### Fomite swabs

Of the 144 samples collected following contact with infected pigs but prior to biosecurity procedures, 11 (8%) were low level positives via RRT-PCR (Table 4). All samples collected following contact with infected pigs but prior to biosecurity procedures from gloves and bouffant cap/respirator were negative, while 7/36 (19%) and 4/36 (11%) samples collected from boots and coveralls were low level positives, respectively. All RRT-PCR low level positive fomite swabs were virus isolation negative. All four personnel had at least one low level positive fomite sample following contact with infected pigs during the movement period with 1, 4, 3, and 3 samples low level positive from personnel A, B, C, and D respectively. Following biosecurity procedures practiced by personnel C and D in the MB replicates, all fomite samples (n = 72) were negative via RRT-PCR.

**Table 4:** Fomite swab results following contact with infected pigs and prior to biosecurity measures.

	Personnel*			
Movement event	A (LB-2)	B (LB-1)	C (MB-2)	D (MB-1)
1				
2			Boots (39)	Boots (39)
3				
4			Boots (39)	
5		Coveralls (39)		
6	Coveralls (38)**	Coveralls (39)	Boots (39)	Boots (37)
7		Boots (38), Coveralls (39)		Boots (40)
8				
9				

* All samples not listed were negative via RRT-PCR.

** ( ) RRT-PCR Ct value

### Nasal swabs

#### Infected group (I)

Influenza virus RNA was detected via nasal swab sampling from all pigs in group I at least once (Table 5). Both direct contact sentinel pigs were infected 1 to 2 days after the eight inoculated pigs first tested positive. The average detection period (number of days between the first and last detection of IAV RNA via RRT-PCR from nasal swabs) for animals in group I was 7.3 days (range 6–9 days). During the 5 days in which the 9 movement events took place, 5, 8, 9, 10, and 10 pigs were IAV positive or low level positive via RRT-PCR in the infected group. The subset of nasal swab samples (n = 10) tested by virus isolation were virus isolation positive and titers ranged from 1.47×10^3^ to 6.81×10^4^ TCID_50_/mL.

**Table 5:** Pig RRT-PCR results by day from nasal swab samples.

		Study Day																		
Group	Pig	–5	0	1	2	3	4	5	6	7	8	9	10	11	12	13	14	15	16	17
**I**	I-1	–	–	**+**	**+**	**+**	**+**	**+**	S	S	–	**+**	–	–	N	N	N	N	N	N
	I-2	–	–	–	**+**	–	**+**	**+**	–	S	–	–	–	–	N	N	N	N	N	N
	I-3	–	–	–	**+**	**+**	**+**	**+**	S	**+**	**+**	–	–	–	N	N	N	N	N	N
	I-4	–	–	**+**	**+**	**+**	**+**	**+**	S	S	–	S	–	–	N	N	N	N	N	N
	I-5	–	–	–	**+**	**+**	**+**	**+**	–	S	–	–	–	–	N	N	N	N	N	N
	I-6	–	–	S	**+**	**+**	**+**	**+**	S	**+**	–	–	–	–	N	N	N	N	N	N
	I-7	–	–	**+**	**+**	**+**	**+**	**+**	**+**	S	–	–	–	–	N	N	N	N	N	N
	I-8	–	–	**+**	**+**	**+**	**+**	**+**	S	S	–	–	–	–	N	N	N	N	N	N
	I-9*	–	–	–	–	**+**	**+**	**+**	S	**+**	**+**	S	–	–	N	N	N	N	N	N
	I-10*	–	–	–	–	**+**	**+**	**+**	**+**	**+**	**+**	**+**	S	–	N	N	N	N	N	N
**MB-Rep 1**	MB-1	–	–	–	–	–	–	–	–	–	–	–	–	–	–	–	–	N	N	–
	MB-2	–	–	–	–	–	–	–	–	–	–	–	–	–	–	–	–	N	N	–
	MB-3	–	–	–	–	–	–	–	–	–	–	–	–	–	–	–	–	N	N	–
	MB-4	–	–	–	–	–	–	–	–	–	–	–	–	–	–	–	–	N	N	–
	MB-5	–	–	–	–	–	–	–	–	–	–	–	–	–	–	–	–	N	N	–
**MB-Rep 2**	MB-6	–	–	–	–	–	–	–	–	–	–	–	–	**+**	**+**	**+**	**+**	S	–	N
	MB-7	–	–	–	–	–	–	–	–	–	–	–	**+**	**+**	**+**	**+**	S	–	–	N
	MB-8	–	–	–	–	–	–	–	–	–	–	–	S	**+**	**+**	**+**	S	–	–	N
	MB-9	–	–	–	–	–	–	–	–	–	–	S	**+**	**+**	**+**	**+**	**+**	S	–	N
	MB-10	–	–	–	–	–	–	–	–	**+**	**+**	**+**	**+**	**+**	S	–	–	–	–	N
**LB-Rep 1**	LB-1	–	–	–	–	–	–	–	–	–	–	–	–	–	–	–	–	N	N	–
	LB-2	–	–	–	–	–	–	–	–	–	–	–	–	–	–	–	–	N	N	–
	LB-3	–	–	–	–	–	–	–	–	–	–	–	–	–	–	–	–	N	N	–
	LB-4	–	–	–	–	–	–	–	–	–	–	–	–	–	–	–	–	N	N	–
	LB-5	–	–	–	–	–	–	–	–	–	–	–	–	–	–	–	–	N	N	–
**LB-Rep 2**	LB-6	–	–	–	–	–	–	–	–	–	S	–	**+**	**+**	**+**	S	**+**	–	–	N
	LB-7	–	–	–	–	–	–	–	–	S	**+**	**+**	**+**	**+**	**+**	–	–	–	–	N
	LB-8	–	–	–	–	–	–	–	–	–	–	**+**	**+**	**+**	**+**	S	–	–	–	N
	LB-9	–	–	–	–	–	–	–	–	**+**	**+**	**+**	**+**	**+**	S	–	–	–	–	N
	LB-10	–	–	–	–	–	–	S	**+**	**+**	**+**	**+**	**+**	S	S	–	–	–	–	N
**NC**	NC-1	–	–	N	–	N	–	N	N	N	N	–	N	N	N	N	–	N	N	–
	NC-2	–	–	N	–	N	–	N	N	N	N	–	N	N	N	N	–	N	N	–
	NC-3	–	–	N	–	N	–	N	N	N	N	–	N	N	N	N	–	N	N	–
	NC-4	–	–	N	–	N	–	N	N	N	N	–	N	N	N	N	–	N	N	–
	NC-5	–	–	N	–	N	–	N	N	N	N	–	N	N	N	N	–	N	N	–
**Group**	**Pig**	–**5**	**0**	**1**	**2**	**3**	**4**	**5**	**6**	**7**	**8**	**9**	**10**	**11**	**12**	**13**	**14**	**15**	**16**	**17**
		**Study Day**																		

* Direct contact sentinel.

(–)  =  Negative (Ct value >40).

(+)  =  Positive (Ct value ≤35).

S  =  Low level positive or suspect (Ct value >35 and ≤40).

N  =  Not tested.

#### Low biosecurity sentinel group (LB)

Influenza virus was not detected via nasal swabs from pigs in LB replicate 1; however, all 5 pigs in LB replicate 2 were infected with IAV. The average detection period for animals in LB replicate 2 was 6.4 days (range 5–8 days).

#### Medium biosecurity sentinel group (MB)

Influenza virus was not detected via nasal swabs from pigs in MB replicate 1; however, all 5 pigs in MB replicate 2 were infected with IAV. The average detection period for animals in MB replicate 2 was 5.6 days (range 5–7 days).

#### Negative control group (NC)

Influenza virus was not detected via nasal swab samples from pigs in the negative control group.

### Serology

**Figure 3:**
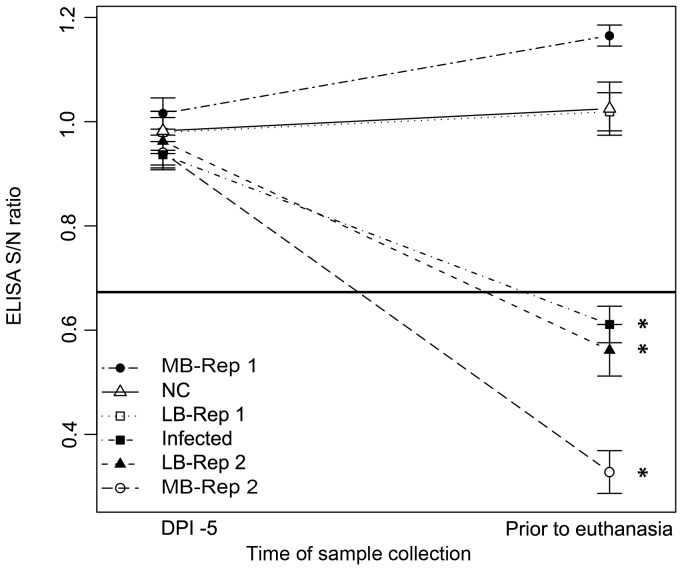
Influenza A Multiscreen ELISA S/N ratios (±SE) by experimental group and replicate. The black horizontal line represents the cutoff value (≤0.673 is considered positive). *Significantly lower ELISA S/N ratios prior to euthanasia compared to -5 DPI (P<0.05).

All pigs were seronegative via ELISA (S/N ratio > 0.673) at the beginning (-5 DPI) of the study (Figure 3). Pigs in LB replicate 1, MB replicate 1, and the NC group were seronegative via ELISA prior to euthanasia, while ELISA S/N ratios were significantly lower (positive) in pigs from LB replicate 2 (P  =  0.002) and MB replicate 2 (P  =  0.001) prior to euthanasia compared to paired ELISA S/N ratios at -5 DPI. In addition, ELISA S/N ratios were significantly lower (positive) in pigs from group I prior to euthanasia compared to paired ELISA S/N ratios at -5 DPI (P <0.0001).

### Genetic sequencing (HA1)

HA1 gene sequencing of one positive matrix RRT-PCR positive sample from each infected pig in the LB and MB groups (n = 10), the inoculum (n = 1), and two RRT-PCR positive samples from two different pigs in group I revealed that all HA1 gene sequences shared greater than 99.7% nucleotide similarity.

## Discussion

Influenza virus transmission routes within and between pig populations have not been fully elucidated. In addition, experimental studies evaluating the entire indirect (fomite and/or contaminated personnel) transmission chain from influenza infected to susceptible hosts are scarce across all species, including humans. This experimental study provides evidence that fomites can be contaminated with IAV following interaction with infected pigs, IAV can be transported via fomites to non-contiguous groups of sentinel pigs, and that sentinel pigs can become infected with IAV following the contamination and transport of fomites by personnel. Furthermore, additional biosecurity measures did not prevent transmission in one of two replicates.

The first necessary step in an indirect (fomite) transmission chain is an infected population capable of contaminating fomites, such as coveralls and boots. A strength of this study was the presence of many acutely infected pigs during the nine movement events. This allowed personnel to have extended, close contact with known infected pigs for the duration of the study. At 2 DPI (movement events 2 and 3), all nasal swab samples from inoculated pigs were RRT-PCR and virus isolation positive. In addition, at 5 DPI (movement events 8 and 9), nasal swab samples from the direct contact sentinels were RRT-PCR and virus isolation positive. This confirms that not only were pigs in the infected group influenza virus positive, but they were also shedding infectious virus.

Contact with infected pigs resulted in 7/36 (19%) and 4/36 (11%) swab samples collected from boots and coveralls respectively as low level positives for IAV via RRT-PCR. Virus isolation was attempted from RRT-PCR low level positive fomite samples; however, all samples were virus isolation negative. While the sensitivity of the sampling method is unknown and difficult to quantify in this setting, IAV RNA was present on boots and coveralls of personnel shortly after contact with infected pigs. The swab based sampling method used in this study may have impacted the recovery rate of IAV from certain materials, such as those that are more absorbent (e.g. N-95 mask and bouffant cap). Enhanced detection methods have been used to successfully recover influenza virus from absorbent surfaces [10] and use of such methods may have resulted in greater recovery of IAV from clothing and PPE used in this study. The number of positive fomite samples was also likely dictated by the interaction preference of pigs as boots and coveralls were more accessible to pigs during this study. A unique aspect of this study was the subsequent exposure of sentinel pigs to these materials as the ultimate measure of IAV infectivity and transmission. The contamination of hands, oral mucosa, and nasal mucosa of personnel following contact with infected pigs could not be assessed in this study as necessary personnel protective equipment (gloves and N-95 respirator) was worn by personnel at all times to prevent interspecies transmission.

While the role of fomites concerning IAV transmission has been studied previously, often the transmission chain is ended following the contamination of fomites or assessing the transmission chain further is not possible and inferences on the likelihood of transmission are made based on the duration or frequency of IAV presence on specific materials [10], [12], [18]. This study was able to continue the transmission chain by exposing sentinel pigs to recently contaminated fomites. With all other known transmission routes outside of fomites controlled for and minimized (e.g. direct pig contact, infected personnel, aerosol) the role of fomites via movement of personnel could be assessed specifically.

Following the first detection of IAV via daily sampling from one pig in the LB and MB replicates, all 5 pigs in each replicate became infected over time. Based on the timing of IAV detection in the LB and MB groups and the 1–2 day latent period observed in group I following experimental inoculation, it appears that a small subset of pigs may have been infected due to IAV exposure via fomites and the remainder was likely due to pig to pig transmission. Differences in pig behavior and interaction with the study personnel and exposure dose may explain why all pigs did not become infected at the same time. Movement events from group I to groups LB and MB did not occur after DPI 5. Therefore, it is unlikely that new infections ≥ 8 DPI were due to exposure to contaminated fomites from group I. This observation along with the low frequency of virus isolation negative, but RRT-PCR low level positive fomite samples following contact with infected pigs may indicate that the amount of infectious IAV on fomites was low. In addition, one of the LB replicates remained IAV negative following nine personnel movement events over a five day period from a room with 5, 8, 9, 10, and 10 infected pigs each day and evidence of IAV RNA on coveralls and boots transported to the respective room. The LB replicate that did become infected was visited by person A who wore cloth coveralls during the nine movement events in contrast to person B who wore Tyvek® coveralls. Influenza virus RNA was detected from coverall swab samples of personnel A and B, while only the sentinel pig room visited by person A became infected. The coverall material may have impacted the survival of IAV and the resultant transmission results; however, all other equipment worn by study personnel was the same and IAV RNA was also detected from the boot swab samples of person B. Additional research would be required to assess the potential impact of different coverall types on transmission.

The frequency at which infection events would take place in this setting under the specific biosecurity procedures cannot be assessed due to limited replication. Of particular interest was the infection of pigs in one of the medium biosecurity replicates. Due to the limited replication in this study, we cannot conclude that the biosecurity measures practiced reduced or increased the frequency of IAV infection. In this experimental setting, the medium biosecurity groups were placed either between or in an adjacent room to the low biosecurity groups. While it is unlikely that a higher containment population would be placed directly adjacent to or between lower containment populations in a field setting, the procedures practiced in this study and the experimental layout avoided the potential transmission from LB to MB replicates. For example, the shared animal isolation hallway was only entered after personal hygiene measures were performed and contaminated outer clothing and PPE were removed in the anteroom. In addition, all rooms were separately ventilated as described in the methods section to prevent aerosol transmission between rooms. It has been well documented in experimental and observational studies that certain biosecurity measures may prevent or limit the spread of pathogens in pigs such as PRRSv, *Escherichia coli*, and *Mycoplasma hyopneumoniae* [8], [9], [19], [20]. In humans, a review of physical measures including hand washing and mask use to reduce respiratory virus transmission indicated that these measures were also effective and should be implemented and assessed further [21]. Knowing that biosecurity and hygiene measures can be effective in preventing or limiting spread of pathogens, the results of this study do not significantly alter current recommendations. However, this study does highlight the potential for IAV transmission by fomites in the presence of biosecurity and hygiene measures.

Personnel did not shower or change clothes worn underneath coveralls between movement events from group I to the MB replicates. Therefore, it is possible that contamination of clothes worn underneath coveralls or areas of the skin that were not washed could have been contaminated with IAV over the 9 movement events. While assessing the mechanical transmission of enterotoxigenic *Escherichia coli*, transmission was prevented when people showered and wore clean outerwear after interacting with infected pigs; however, transmission was not fully prevented via hand washing and wearing clean outerwear [8]. Most importantly, our study indicates that IAV can be transmitted via fomites and result in the infection of previously negative pigs.

Influenza virus is a common respiratory pathogen in pigs and this study illustrates that fomites can transmit IAV from infected to susceptible populations. While the frequency of this event cannot be determined, this transmission route should be taken into account under existing comprehensive biosecurity protocols in the field. In addition to the fomites assessed in this study, influenza viruses have been detected and shown to remain viable on various other fomites. For example, viable influenza virus has been recovered from stainless steel and plastic surfaces, paper tissue, and banknotes following contamination and has been detected on fomites in human settings, such as day care centers [11], [12], [22]. While fomites can be contaminated, recent work has shown that influenza virus may not survive for a long period following natural contamination [10]. Movement to sentinel rooms following contact with infected pigs was immediate in this study, and thus long term survival of IAV on fomites was not necessary for transmission. Short time intervals between contact with infected individuals and susceptible individuals may occur in many settings, including within day care centers and pig barns, indicating that transmission can take place even though the survival of IAV may be short.

Influenza virus infections are common in both pig and human populations and the pandemic 2009 H1N1 and H3N2 variant (H3N2v) viruses have recently highlighted the interspecies transmission potential of IAV [7], [23]. A thorough understanding of critical transmission routes is needed in order to mitigate IAV transmission both within species and between species. This study has confirmed the transmission of IAV by fomites between pig populations in an experimental setting under differing biosecurity measures. Biosecurity and hygiene measures aimed at indirect routes of transmission, including fomites, should be incorporated and further assessed as part of comprehensive biosecurity protocols to prevent IAV transmission.
